# Identification of muscle-specific candidate genes in Simmental beef cattle using imputed next generation sequencing

**DOI:** 10.1371/journal.pone.0223671

**Published:** 2019-10-10

**Authors:** Farhad Bordbar, Just Jensen, Bo Zhu, Zezhao Wang, Lei Xu, Tianpeng Chang, Ling Xu, Min Du, Lupei Zhang, Huijiang Gao, Lingyang Xu, Junya Li

**Affiliations:** 1 Laboratory of Molecular Biology and Bovine Breeding, Institute of Animal Science, Chinese Academy of Agricultural Sciences, Beijing, China; 2 Department of Molecular Biology and Genetics, Aarhus University, Aarhus, Denmark; 3 Department of Animal Sciences, Washington Center for Muscle Biology, Washington State University, Pullman, Washington, United States of America; Universidade Federal de Mato Grosso do Sul, BRAZIL

## Abstract

Genome-wide association studies (GWAS) have commonly been used to identify candidate genes that control economically important traits in livestock. Our objective was to detect potential candidate genes associated mainly with muscle development traits related to dimension of hindquarter in cattle. A next generation sequencing (NGS) dataset to imputed to 12 million single nucleotide polymorphisms (SNPs) (from 1252 Simmental beef cattle) were used to search for genes affecting hindquarter traits using a linear, mixed model approach. We also used haplotype and linkage disequilibrium blocks to further support our identifications. We identified 202 significant SNPs in the bovine BTA4 chromosome region associated with width of hind leg, based on a stringent statistical threshold (p = 0.05/ effective number of SNPs identified). After exploring the region around these SNPs, we found candidate genes that were potentially related to the associated markers. More importantly, we identified a region of approximately 280 Kb on the BTA4 chromosome that harbored several muscle specific candidate genes, genes to be in a potential region for muscle development. However, we also found candidate gene *SLC13A1* on BTA4, which seems to be associated with bone disorders (such as chondrodysplasia) in Simmental beef cattle.

## Introduction

Cattle and other livestock species are economically important in almost all regions of the world [1]. Cattle breeds are continuously being developed to optimize the efficiency of meat production [2] and meat-production traits, controlled by a variety of genes, are among the most important traits selected for in various beef cattle breeding programs [3]. More specifically, cattle breeders desire cattle with more rapid lean muscle growth. The focus on lean muscle is important because consumers today demand leaner meat [4], thus making muscle growth an important economic factor in the beef industry. Therefore, within the past few years, more research has focused on economically important traits affecting the quality of meat production in livestock, particularly on muscle development. However, breeding programs are having difficulty addressing muscle quality problems in livestock, such as poor meat quality in swine, in part due to insufficient information about underlying mechanisms involved with muscle growth and its metabolism [5].

A candidate gene approach, used in genetic association studies for a variety of species [6], might be useful for improving our understanding of genes responsible for regulating traits involved with the underlying metabolism of muscle growth. That is, because candidate genes possess specific biological actions relative to various aspects of a target trait, the candidate gene approach can provide a valuable strategy for detecting the QTL (quantitative trait loci) that controls genetic variation of an important trait [7].

Genome-wide association studies (GWAS) provide a robust approach for identifying genetic variants that exert prominent effects on quantitative traits and complex diseases in humans [8–10] and farm animals [11]. Recently, several association studies related to economically important traits (production, health, reproduction and body conformation) in cattle have been published [12–14]. However, the success of the GWAS approach depends on a variety of factors, such as sample size and the number of genetic variants affecting the trait [15].

Next generation sequencing (NGS), a recently developed method, has been used to incorporate more genetic variants than SNP (single nucleotide polymorphism) arrays. Generally, an NGS dataset exhibits a higher error rate compared to genotyping based on DNA chips, however, this problem can be remedied by imputing a larger number of genetic variants. Applying NGS in association studies offers numerous advantages over traditional DNA sequencing approaches, including higher throughput sequencing, more specific applicability, and higher read quality for sequencing dataset [16]. Using an NGS dataset, Sharma et al. pinpointed 18 mutations associated with Mendelian diseases in Hanwoo cattle [17], whereas 33 genes were identified as being associated with the domestication process [18]. The NGS approach is also useful for investigating genes associated with complex traits. For example, three loci have been identified in the European domestic pigs [19], including the *PLAG1* and *LCORL* genes, both of which are associated with growth rate and meat quality in many livestock species. However, one of the problems associated with using an NGS dataset is capacity of typical computers to calculate marker effects, due to high density of SNPs [20].

Previous studies have found that Chromosomes 2, 3, 6, 14, 20, and 29 exert significant effects (location for various novel QTLs) on important carcass traits such as meat production in cattle [21–25]. In fact, muscle specific genes are distributed over several chromosomes. For example, *Alpha-actin* (*ACTA1*) is located on bovine Chromosome 28 (identified using various analyses [26–27]), whereas bovine Chromosome 5 harbors *Myogenic factor 5* (*MYF5*), first identified by linkage mapping [28]. Using linkage analysis, Charlier et al. identified the locus for double muscling associated with the *MH* gene on bovine Chromosome 2 [29]. The important muscle-specific gene *myogenin* (*MYOG*) was mapped on bovine Chromosome 16 [30]. *Calpain 1* (*CAPN1*) and *calpastatin* (*CAST*) genes, which play important roles in meat tenderness, have been mapped on bovine Chromosomes 29 and 7, respectively [31, 32].

Hind quarter provide the most valuable cuts on the beef carcass and can, therefore, be advantageous specifically for growth and meat leanness traits. The hind quarter include connective tissues, major weight supporting bones such as femur, tibia, and fibula and variety of covering muscle tissues, such as gastrocnemius, biceps femoris, semitendinosus, semimembranosus, vastus lateralis, tensor fascia lata, and gluteus medius. Almost all of these muscles are large skeletal muscles that tend to produce copious amounts of tender meat. Piedrafita et al. reported a close correlation between the dimension of the hind leg and percent muscle [33]. Moreover, a direct association was documented between thickness of hind limb and growth rate of European beef cattle [33].

Many papers have focused on hind legs relative to muscle development, bone disorders, and protein metabolism [34–39]. In this study, we use the GWAS approach to identify candidate genes associated with the width, length, and circumference of hind legs. More specifically, the major aim of this research was to identify markers and QTLs that significantly affect the size of the hind quarter in the Simmental breed. We suspect that various factors could potentially affect lean meat production in the hind leg, hind-leg bone characteristics, and location of fat in the hind leg. We expect our result to elucidate the underlying molecular mechanism involved with hind-leg meat production in Simmental cattle. To our knowledge, our study is the first to apply GWAS analysis to attempt to identify candidate genes responsible for hind leg related traits in Simmental beef cattle.

## Materials and methods

### Ethics statement

We followed guidelines advanced by the China Council on Animal Care. All steps and activities were authorized by the Institute of Animal Science (IAS) and the Chinese Academy of Agricultural Science (CAAS), Beijing, China. Animal examinations followed rules of the China’s Animal Welfare council.

### Animal resources and phenotype data

**Fig 1:**
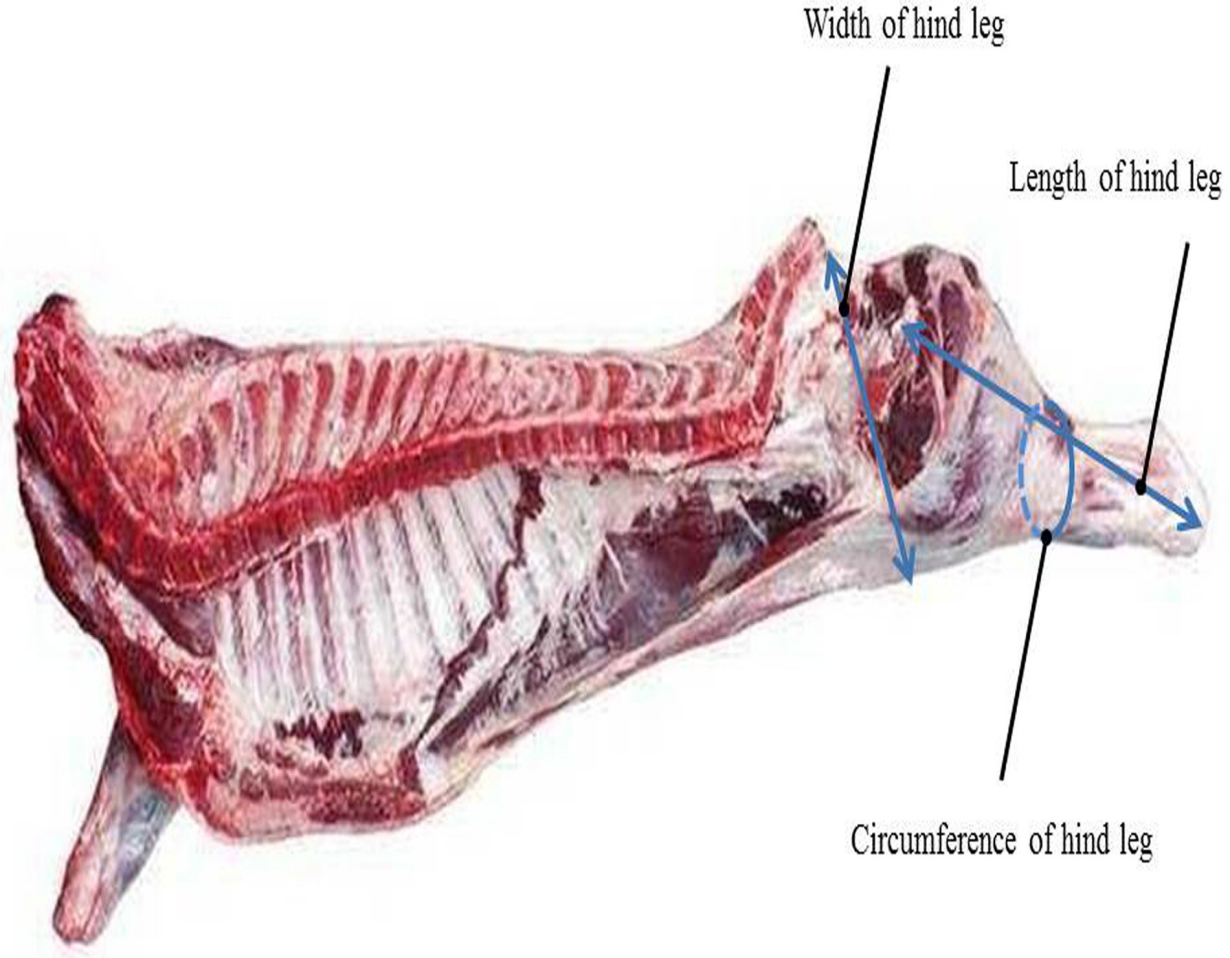
Locations of width, length, and circumference of hind leg measurements for the experimental beef carcasses.

Our population included 1346 Simmental beef cattle born from 2009 to 2015. The animals originated from the Chinese regions of Ulgai, Xilingol League, and Inner Mongolia. After weaning, calves were transferred to the Beijing Jinweifuren farm feedlot. All calves were raised under the same feeding and management protocols. At 16 to 18 months of age, animals were slaughtered via electric stunning. [Due to the long distance from the feed lot to the abattoir (almost 332 km, 10-h drive), all cattle were rested for almost one day before slaughtering them to make sure that they had recovered from traveling and any related stress.] After bloodletting, carcasses were split (cut open) vertically into two equal parts and then weighed. Split carcasses were immediately placed in refrigerators at 4°C until examined traits could be measured (approximately nine hours after slaughtering). Examined traits were measured according to strict guidelines outlined by Institutional Meat Purchase Specifications for fresh beef. Measurements of the hind leg included the width, length, and circumference (Fig 1). Width of hind leg is the straight-line distance from the inside of the tail recess to the leading edge of the thigh (https://www.greatfoodsolutions.com/products/beef-carcass-regular-cut. Accessed 23 NOV. 2018), length of hind leg is the distance from the anterior border of the pubic suture to the midpoint of the ankle joint, and circumference of the hind leg is horizontal circumference at the junction of the femur and tibia. Mean, standard deviation, maximum, and minimum of the measured traits are listed in Table 1.

**Table 1:** Descriptive statistics for hind-leg measurements of experimental animals.

Trait	Mean (cm)	Standard deviation (cm)	Maximum (cm)	Minimum (cm)
The width of hind leg	44.86	4.13	67	25
The length of hind leg	76.78	5.16	92	50
The circumference of hind leg	68.01	16.58	110	22

### Genotype data and quality control of SNP array

We contracted with the Illumina Genome Studio (San Diego, CA) to sample genotypes and analyze SNP chips. To quality control, we removed cattle with high Pi-Hat values and subjected the other cattle to exclusion criteria consisting of SNPs with call rates less than 99%, minor allele frequencies (MAF) less than 5%, and severe deviations from Hardy-Weinberg equilibrium (*P* < 10^−6^). In addition, individuals with >10% missing genotypes were removed from the dataset. All these criteria were processed by PLINK v1.07 [40]. After control quality, 1252 Simmental beef cattle with 671,204 autosomal SNPs remained for subsequent analyses.

### Resequencing

Based on genomic relationships and Pi-Hat values, we chose 44 unrelated Simmental beef cattle for genetic resequencing of whole genome. We used the TIANamp Blood DNA kit (Tiangen Biotech Company Limited, Beijing, China) to construct NGS library, which consisted of choosing DNA with an A260/280 nm absorbance ratio ranging between 1.8 and 2.0. We prepared one library for each animal (n = 44) using an Illumina Hiseq 2500 genome sequencing system (Illumina Inc., San Diego, CA, USA). We obtained 9,621,765,847 reads, which we then subjected to our quality control process. We excluded low-quality reads, such as those containing more than 10% unknown bases, more than 10% mismatches, or more than 50% low-quality bases. We also removed putative PCR duplicates produced by PCR amplification in the library construction process.

**Fig 2:**
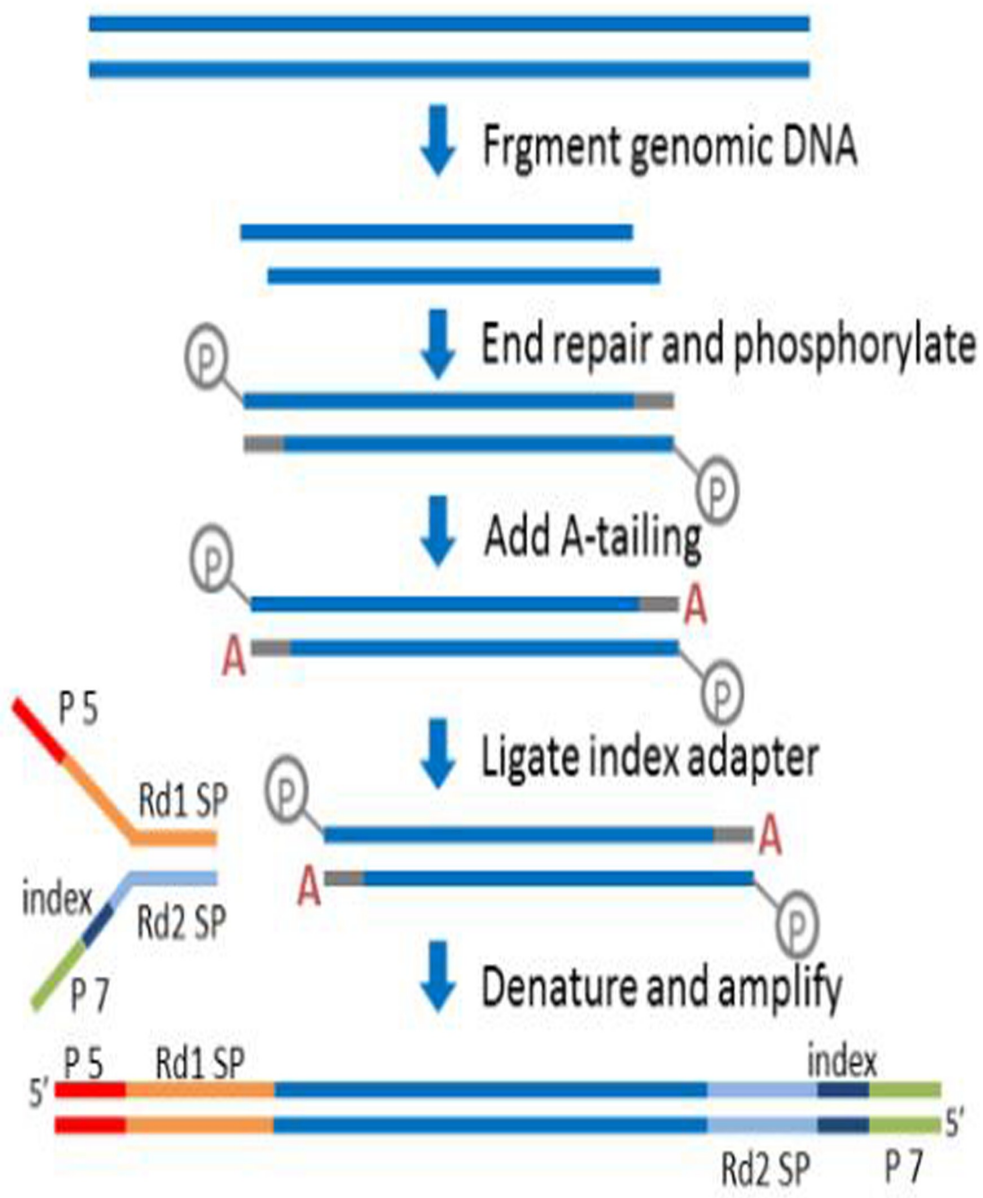
Library construction plot. The plot was provided by Genome Sequencing company Novogene (https://en.novogene.com).

We used 1.5 μg of DNA per sample as input material for each sample. We generated sequencing libraries using the Truseq Nano DNA HT sample preparation Kit (Illumina Inc., San Diego, CA, USA) following manufacturer’s recommendations and added index codes to attribute sequences for each sample. We fragmented each DNA sample with sonication to 350 bp, then DNA fragments were end polished, A-tails added, and ligated with the full-length adapter for Illumina sequencing with further PCR amplification (Fig 2). We purified PCR products with an AMPure XP system and analyzed library size distributions with an Agilent 2100 Bioanalyzer and then quantified them using real-time PCR. After implementing our quality control procedures, we obtained 9,584,920,309 reads. Sample reads and quality scores (Q), which represents the quality of sequencing data [41], are listed in S1 Table. The average sequencing depth for each sample was about 20X with a range of 17X to 25X (X represents a number of times which each base is sequenced).

### Imputation of sequence variants

Sequence SNPs with MAF more than 0.05 were subjected to imputation. Based on 21,043,398 sequence variants in the 44 genetically sequenced cattle, we performed imputation using BEAGLE v4.1 [42] with its default parameter settings. Beagle is based on an algorithm using population-based information to infer haplotypes and missing genotypes. We coded genotypes for imputed sequence variants as 0 (for homozygotes), 1 (for heterozygotes), or 2 (for alternative homozygotes). R-sq. data (showing imputation quality [43]) larger than 0.1 were retained for association studies. Following imputation, we obtained 12,468,401 markers for Chromosomes 1–29.

### Statistical analysis

We used a general mixed linear model, determined as follows,

$$y=\mu +Xb_{i}+mjb_{j}+Zu+e$$

Where, *y* is the vector of phenotypic values for traits of interest and μ is the population mean. We used single marker regression with two types of fixed effect variables, including *b*_*i*_, representing the vector corresponding to the nuisance fixed effect (including birth year, sex, weight, number of fattening days) and *b*_*j*_, the SNP effect and *m*_*j*_ representing the vectors of the *i*^*th*^ marker. Variable *u* is the polygenic effect with the assumption of *N* (*0*, *σ*^*2*^*K*), where *K* is defined as the kinship matrix (all markers on autosomal chromosomes were used except for those markers which were under investigation in *b*_*j*_) and *σ*^*2*^ is the additive genetic variance. Variable *X* is the incidence matrix relating the phenotypic observations to fixed effects, whereas *Z* is the matrix relating the phenotypic observations to polygenic effects. Variable *e* is a vector for random residual effects with the assumption that ${V(e)=I\sigma }_{e}^{2}$, where *I* represents the identity matrix and $\sigma_{e}^{2}$ represents residual variance.

We used the GenABEL v1.8–0 [44] algorithm in the R package to identify the statistical significance of each SNP; based Bonferroni corrected P-values of 0.05 divided by the effective number of SNPs. We calculated the t statistic to determine the P-values of each SNP. We used the UCSC genome browser (http://www.genome.ucsc.edu) to search for candidate genes. We estimated haplotype blocks by PLINK v1.07 [41] using default commands (*plink—bfile mydata—blocks*) which calculated LD (linkage disequilibrium) for SNPs within 200 kb in length. To check the association of each haplotype in block, following parameters were used: *plink—bfile mydata—hap plink*.*blocks—hap-freq* [41]. The Haploview v4.2 algorithm [45] was applied to detect haplotype blocks illustrated for each chromosome.

### SNP distribution

One problem associated with analyzing NGS data is the high throughput density of SNPs, which makes it difficult to compute p-values of the SNPs with computers typically accessible by researchers [20]. To address this problem, we divided the bovine chromosomes into three segments: Segment 1 consisting of Chromosomes 1–10 (5,830,727 SNPs), Segment 2 consisting of Chromosomes 11–20 (4,063,690 SNPs), and Segment 3 consisting of Chromosomes 21–29 (2,573,984 SNPs). We applied different p-value thresholds for each segment due to the different numbers of SNPs in each segment.

## Results

### Association analysis

**Fig 3:**
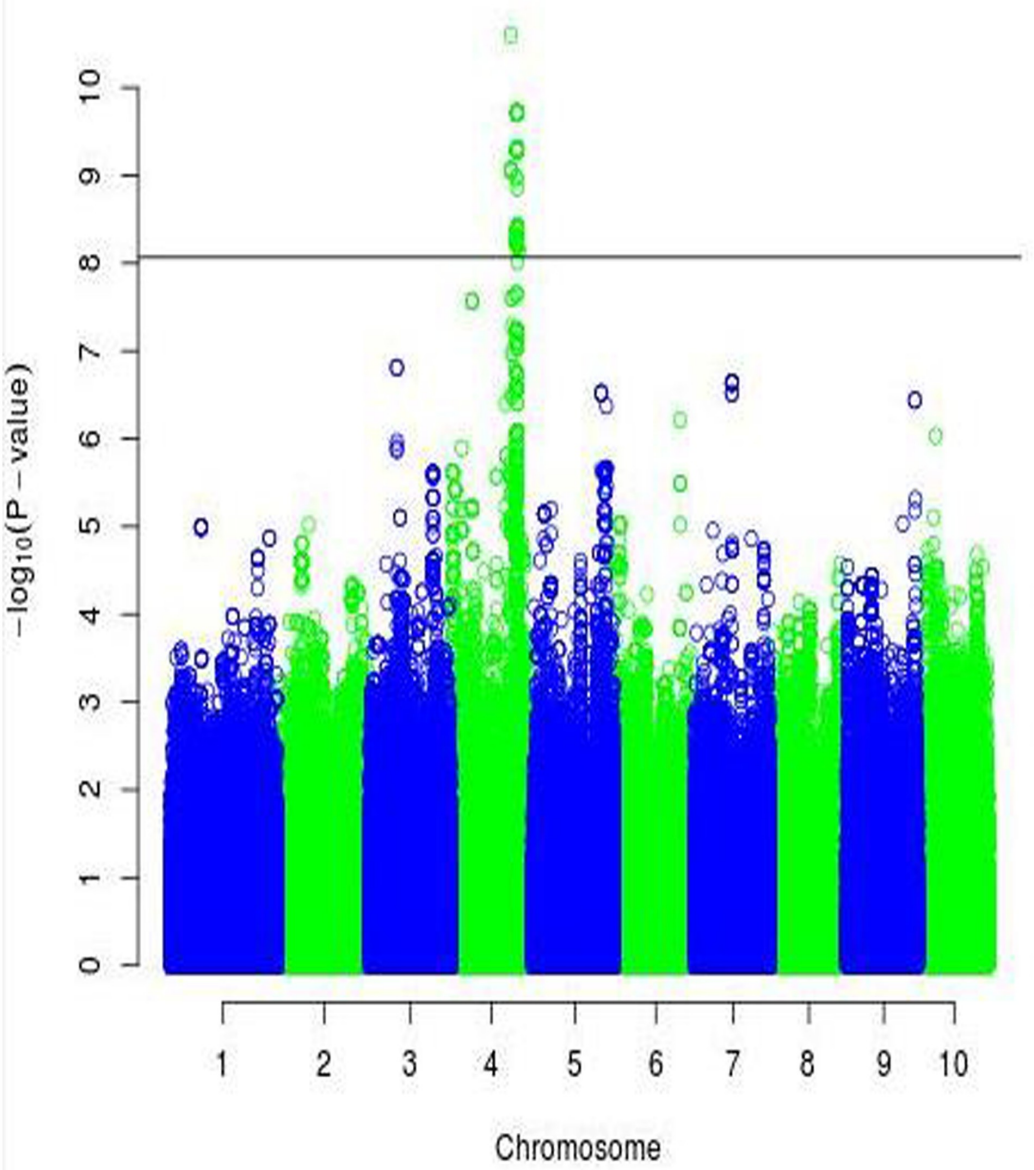
Manhattan plot of -log10 (p) for the region associated with the width of hind leg. The horizontal line represents the assigned threshold of significance.

**Fig 4:**
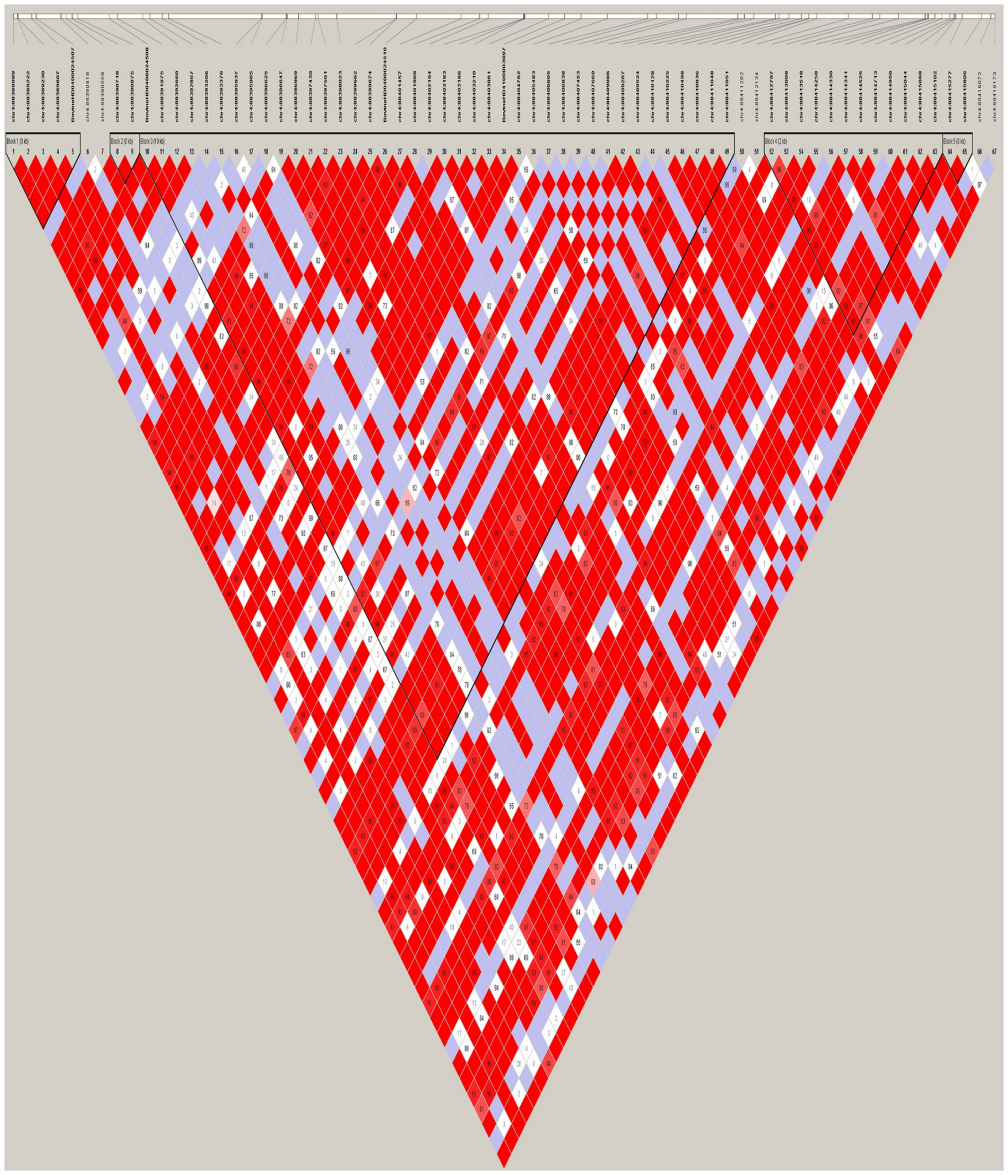
Linkage disequilibrium plot. The high LD block spans 19 kb, which harbors the most significant SNP for width of hind leg.

To identify more reliable QTLs and candidate genes, we set a stringent threshold to each segment (p = 0.05/ effective number of SNPs) [i.e., Segment 1 (p = 8.58×10^−9^), Segment 2 (p = 1.24×10^−8^), and Segment 3 (p = 1.95×10^−8^)]. Fig 3 illustrates the Manhattan plot for the significance of Segment 1 (associated with the width of the hind leg). Surprisingly, we identified 202 SNPs in the BTA4 chromosome that surpassed our established significance threshold, thus making them potential candidates for QTLs (related to the width of the hind leg). We divided these significant SNPs into Sub-sections 1–5 based on their physical locations: (1) 88 Mb (7 SNPs), (2) 96 Mb (18 SNPs), (3) 97 Mb (115 SNPs), (4) 98 Mb (61 SNPs), and (5) 100 Mb (1 SNP). We identified three candidate genes (*IQUB*, *NDUFA5*, and *ASB15)* for Sub-section 1, which spanned with a range of about 322 kb. These three genes were located in close vicinity to one another, totally separated by only 30 kb. Of the seven associated SNPs in Sub-section 1, two SNPs (*chr4*:*88718818* and *chr4*:*88724320*) were located in the *ASB15* gene region, and two SNPs (*chr4*:*88581497* and *chr4*:*88619410*) were located in the *IQUB* gene region. Moreover, by expanding the region about 45 kb downstream of the highest associated SNP with width of hind leg (*chr4*:*88402194*), we found candidate gene *SLC13A1*. We also identified two more candidate genes, *LMOD2* and *WASL*, approximately 50 kb upstream of gene *ASB15*. Haplotype analysis showed both flanking SNPs in Sub-section 1 were located in two haplotype blocks, each spanning 27 kb. Fig 4 Illustrates Linkage disequilibrium plot for the most significant SNP (*chr4*:*88402194*) (*P* = 2.52 x 10^−11^) located in high LD block.

**Fig 5:**
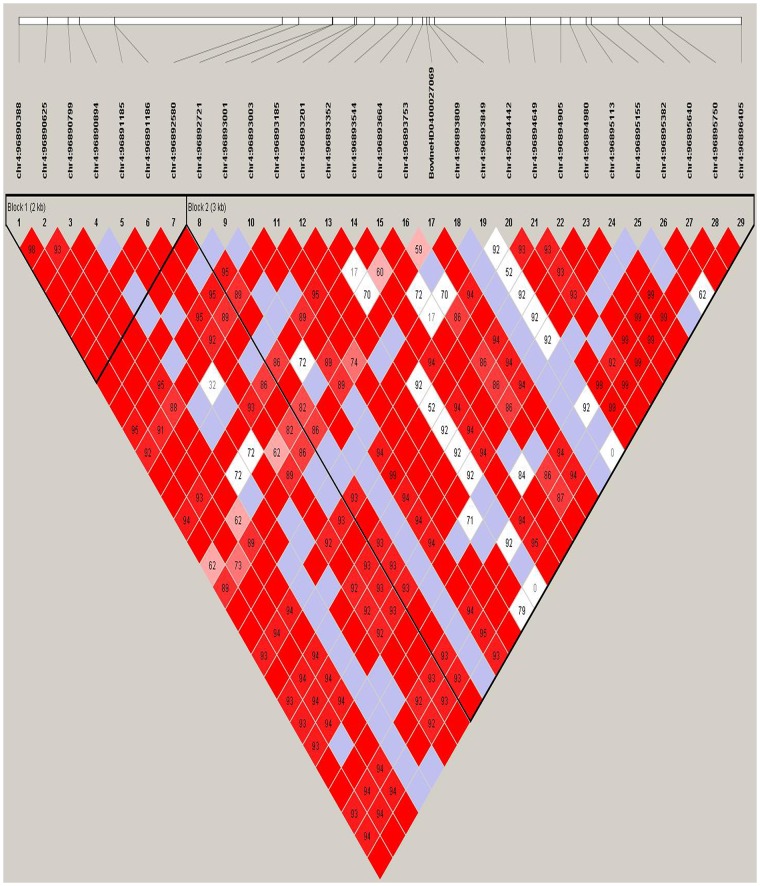
Linkage disequilibrium plot. The plot reveals a high LD block spanning 3 kb around the associated SNP *chr4*:*96893352* in Sub-section 2.

For Sub-section 2, we obtained one candidate gene (*PLXNA4*) with 18 significant SNPs. The distance between these 18 SNPs was about 130 kb and interestingly, all these SNPs were located within the *PLXNA4* gene (464 kb long). Most SNPs were located in or near high LD blocks, such as SNP *chr4*:*96893352*, located in the 3-kb-high LD block shown in Fig 5.

Sub-section 3 corresponded to 115 associated SNPs spanning about 90 kb. After we noticed there was no candidate gene between the flanking SNPs of *chr4*:*97748185* (p = 1.93 x 10^−10^) and *chr4*:*97657432* (p = 6.16 x 10^−9^), we investigated 100 kb downstream and upstream of both flanking SNPs. For SNP *chr4*:*97748185*, we discovered the candidate gene *EXOC4*, located at almost 50 kb upstream of the SNP. Moreover, for SNP *chr4*:*97657432*, we identified the candidate gene *CHCHD3* about 50 kb downstream of the SNP. Although there was no candidate gene between the above two markers, we found about 10 haplotype blocks totally spanning 45 kb, which suggests the importance of the 115 markers identified in this region of the genome.

The *EXOC4* gene was also located in the Sub-section 4, which spanned around 26 kb of BTA4 chromosome. This candidate gene is one of the largest genes, with an approximate length of 800 kb. More importantly, the *EXOC4* gene harbored all significant SNPs in Sub-section 4 (61 SNPs) and most of these SNPs which totally corresponded to 32 kb haplotype blocks were near or within high LD blocks. In addition, after searching for more candidate genes, we noticed the location of the micro-RNA *MIR2423* (73 bp), which was almost 55 kb upstream from SNP *chr4*:*98014505*.

There was only one SNP (*chr4*:100895756) associated with fifth sub-section of Segment 1. When investigating the region around this SNP, we identified a very potentially important candidate gene for Simmental beef cattle, called *MTPN*, located approximately 250 kb downstream from the SNP. (We are in the process of validating this gene). The width of statistically significant hind leg SNPs are listed in S2 Table.

We found only one SNP (*chr5*:*91327124*) in BTA5 chromosome associated with the circumference of hind leg, (p = 1.23 x 10^−9^). After exploring the region around the SNP, we did not find any candidate genes in close proximity. Even when we tolerated a less stringent threshold, we could not identify any significant SNPs associated with hind leg length.

## Discussion

Genome-wide association studies (GWAS) have been applied widely for plants [46] and animals [24]. This method has produced immense strides in elucidating molecular mechanism underlying commercially desirable traits in livestock, such as in beef and dairy cattle [14, 47–52]. In other words, by facilitating the identification of candidate genes with GWAS, breeders are more able to address problems associated with breeding programs (i.e., selecting animals with higher production) and improve desirable traits. Another advantage of GWAS is that entire genomes can be investigated for complex traits examining haplotype blocks and linkage disequilibrium data among genetic markers. Therefore, GWAS is a powerful method for identifying quantitative trait loci and regions that harbor causative genes [53–55]. We expect that the next generation sequencing approach will rapidly improve resequencing procedures for identifying enormous numbers of SNPs, which will in turn enable researchers to identify rare genetic variants and more intensely investigate basic and important mechanisms underlying genetic traits [16].

Interestingly, among the candidate genes located in the BTA4 chromosome, most seem to be muscle-related. *ASB15* is a conserved gene expressed mostly in the musculoskeletal system. The gene *ASB15* is involved in a variety of cell processes, such as proliferation and differentiation [56–58]. *ASB15* also plays a major role in signal transduction pathways encouraged by β-adrenergic agonists (BA) receptor compounds in hypertrophied skeletal muscles [59].

McDaneld et al. found that the *ASB15* gene induces protein turnover and synthesis and so stimulates myoblast differentiation and muscle cell development [60]. In addition, McDaneld and Spurlock reported that *ASB15* alters C2C12 mouse myoblast differentiation via mediation of the PI3K/Akt signal transduction pathway [61]. *NDUFA5* is expressed in a variety of tissues, including in skeletal muscle [62]. This gene, which is involved in transportation of electrons in mitochondria, and is also associated with autism in humans [63]. When conducting miRNA microarray analysis, Seong et al. showed an association between *NDUFA5* and marbling score in Hanwoo cattle [64]. When the amount of feed for Holstein-Friesian bulls was restricted, expression of the *NDUFA5* gene increased and energy production was enhanced [65].

Several studies have proven the existence of *LMOD2* mRNAs in striated muscle [66–68]. In addition, knocking out the *LMOD2* gene can shorten thin filament (actin) prior to dilated cardiomyopathy [69]. Similarly, Yuen et al. showed that the *LMOD2* gene is involved in the regulating the length of thin filaments [70]. After gene expression validation, the *LMOD2* gene has also been shown to be related with muscle development in cattle [71].

In our study, the genes *ASB15*, *NDUFA5*, and *LMOD2* were surrounded by *IQUB* and *WASL* genes (both in close proximity). Furthermore, it has been shown in a cancer research that knockdown of *IQUB* gene can prevent *c-myc* expression [72] (c-*myc* is a muscle regulator gene which is involved with proliferation and differentiation of muscle myoblast).

We also noticed the association of the *WASL* gene which act in the cytoskeleton [73], which is a complex network of cells playing a role in muscle contraction. Taken together, due to all mentioned genes above are related with muscle (either connected with muscle structural proteins or muscle development) we hypothesize that the chromosome region on BTA4 (88 Mb spanning about 280 kb), which supports five closely located genes, is a candidate region for skeletal muscle development genes in Simmental beef cattle. Although the functions of some of the genes occurring in this region have been identified, more research is needed to verify the identity of other candidate genes, specifically regarding their association with differentiation and proliferation of skeletal muscle cells.

Humans and several other species, including *Bos taurus*, can contract a serious genetic disease called chondrodysplasia (dwarfism) which causes abnormality in bone shape and skeleton structure [74]. A mutation (deletion) in gene *SLC4A2* is related to osteoporosis in Red Angus cattle [75]. Similarly, in Texel sheep a mutation in the *SLC13A1* gene (1-bp deletion of T [*g*.*25513delT*] at the 107 bp position of exon 3) is directly associated with chondrodysplasia [76]. Thus, it appears that mutations in solute carrier families (e.g., in genes *SLC4A2* and *SLC13A1*), can cause bone disorders (e.g., chondrodysplasia) in livestock. In our research, we located the *SLC13A1* gene downstream from the most associated SNP (*chr4*:*88402194)* with width of hind leg. Therefore, we speculate that a mutation in gene *SLC13A1* may be responsible for bone disorders, especially in Simmental beef cattle. Hopefully, this insight can be used to help the cattle industry improve animal welfare and reduce economic losses.

We found that the gene *PLXNA4* (at 96 Mb) harbored 18 statistically significant SNPs. This gene belongs to the *plexin-A* subfamily of genes associated with the development of the nervous system. Moreover, the *PLXNA4* gene has the ability to regulate diverse processes, such as cell migration and neural growth cones (a domain with cytoskeletal instructions for actin) [77]. In humans, this gene plays a crucial role in denervation of skeletal muscles in motor neurone disease (MND) [78]. The function of this gene in cattle skeletal muscle is completely unknown; however, we hypothesize that the *PLXNA4* gene has a potential role in the development of skeletal muscle. However, more research is needed on functional relationships between the gene and skeletal development to test our hypothesis.

In the third sub-section we examined (at 97 Mb), we did not find any candidate genes from associations of SNPs with our measured traits. However, after exploring regions flanking SNPs, we identified two candidate genes (*CHCHD3* and *EXOC4)*. We also identified the *EXOC4* gene in fourth sub-section of segment 1 we examined (at 98 Mb). Interestingly, all associated SNPs (n = 61) in sub-section 4 were located within this gene.

The *CHCHD3* gene is primarily expressed in mitochondria, especially in skeletal muscles [79]. Darshi et al. elucidated that the *CHCHD3* gene is essential for the formation of crista structure and in cellular metabolism [80], whereas Fan et al. suggested the potential relationship of this gene with back fat in pigs [81]. However, the function of the *CHCHD3* gene is almost unknown in cattle and so further research is needed to substantiate the association of this candidate gene with skeletal muscle and fat development.

The *EXOC4* gene which can cause schizophrenia in humans [82], is crucial for producing insulin and involved in protein and hormone metabolism. A few studies suggest an association between the *EXOC4* gene and meat quality in pigs and in Australian beef cattle. For example, Welzenbach et al. showed that the *EXOC4* gene may be a candidate gene associated with rate of drip loss in pigs [83]. However, more research is needed to identify the association between this candidate gene and meat quality in Simmental beef cattle.

In the sub-section 5 of Segment 1, we identified a very promising candidate gene (*MTPN*) related to skeletal muscle development, but this gene requires further validation. We also found a suite of statistically significant SNPs associated with the width of hind leg, providing candidate genes related to muscle development in the BTA4 chromosome. Our study highlights the possible role of the BTA4 region in molecular mechanism of muscle in Simmental cattle. Most of information we obtained from our research can be applied to breeding programs and thus help clarify mechanisms underlying the establishment of economically important traits in cattle.

## Supporting information

S1 Table Total reads of 44 samples (library) and their quality scores for next generation sequencing. (XLSX)

S2 Table Associated SNPs identified for width of hind leg trait. The GWAS results of width of hind leg containing associated SNPs, p-values, and located gene(s).
(XLSX)
